# Blockchain-Assisted Privacy-Preserving and Context-Aware Trust Management Framework for Secure Communications in VANETs

**DOI:** 10.3390/s23125766

**Published:** 2023-06-20

**Authors:** Waheeb Ahmed, Wu Di, Daniel Mukathe

**Affiliations:** School of Computer Science and Technology, Dalian University of Technology, Dalian 116024, China

**Keywords:** blockchain, authentication, context awareness, trust management, vehicular ad hoc network (VANET)

## Abstract

Vehicular ad hoc networks (VANETs) are used for improving traffic efficiency and road safety. However, VANETs are vulnerable to various attacks from malicious vehicles. Malicious vehicles can disrupt the normal operation of VANET applications by broadcasting bogus event messages that may cause accidents, threatening people’s lives. Therefore, the receiver node needs to evaluate the authenticity and trustworthiness of the sender vehicles and their messages before acting. Although several solutions for trust management in VANETs have been proposed to address these issues of malicious vehicles, existing trust management schemes have two main issues. Firstly, these schemes have no authentication components and assume the nodes are authenticated before communicating. Consequently, these schemes do not meet VANET security and privacy requirements. Secondly, existing trust management schemes are not designed to operate in various contexts of VANETs that occur frequently due to sudden variations in the network dynamics, making existing solutions impractical for VANETs. In this paper, we present a novel blockchain-assisted privacy-preserving and context-aware trust management framework that combines a blockchain-assisted privacy-preserving authentication scheme and a context-aware trust management scheme for securing communications in VANETs. The authentication scheme is proposed to enable anonymous and mutual authentication of vehicular nodes and their messages and meet VANET efficiency, security, and privacy requirements. The context-aware trust management scheme is proposed to evaluate the trustworthiness of the sender vehicles and their messages, and successfully detect malicious vehicles and their false/bogus messages and eliminate them from the network, thereby ensuring safe, secure, and efficient communications in VANETs. In contrast to existing trust schemes, the proposed framework can operate and adapt to various contexts/scenarios in VANETs while meeting all VANET security and privacy requirements. According to efficiency analysis and simulation results, the proposed framework outperforms the baseline schemes and demonstrates to be secure, effective, and robust for enhancing vehicular communication security.

## 1. Introduction

Road accidents are one of the main causes of death on the roads and are becoming more frequent. Every year, millions of people worldwide die in traffic accidents [1]. Additionally, these accidents cause traffic congestion [2], loss of property, lost working hours, and high fuel consumption [3]. It has been shown that most accidents can be avoided by warning drivers one half second in advance [4]. Vehicular ad hoc networks (VANETs) have been developed recently to reduce accidents, improve traffic efficiency and road safety, and enhance user comfort [5]. VANETs enable vehicles to communicate with other vehicles via Vehicle-to-Vehicle (V2V) communication, and to communicate with roadside units (RSUs) via Vehicle-to-Infrastructure (V2I) communication, to exchange information and inform drivers about road hazards. In contrast to traditional networks, VANETs are more vulnerable to a variety of attacks from malicious vehicles due to their unique characteristics, which include high mobility, dynamic topology, volatility, and open wireless communication channels. Malicious vehicles/attackers may intercept and alter the content of received messages before forwarding them, obtain sensitive and private information from these messages to track the sender vehicles’ routes, or create traffic illusions by disseminating false/bogus messages and causing the drivers to make incorrect and life-threatening decisions. Consequently, road safety, traffic efficiency, and the performance of the network can be adversely affected by malicious vehicles and their false/bogus messages. Therefore, a key challenge to the actual deployment of VANETs is communication security [6,7,8]. To provide secure communication for message propagation, the network must meet VANET users’ most important requirements, which are privacy, security, and trust [9]. Existing authentication schemes are designed to satisfy most of the security and privacy requirements of VANETs. These schemes are used to authenticate the network nodes and ensure that exchanged messages are sent by registered vehicles and not modified during transmission. However, authenticated vehicles may send false/bogus messages which are authenticated without being detected. Consequently, existing authentication schemes are not able to ensure a trusted communication environment in VANETs by preventing authenticated vehicles (insider attackers) from sending false/bogus messages in the network. Therefore, trust is introduced as essential and an important component for enhancing the security of vehicular communications [9]. Trust management approaches are adopted to evaluate the trustworthiness of the sender vehicles and their messages, and to identify and revoke those insider attackers/malicious vehicles and their bogus messages [10,11]. Although existing trust management schemes are designed to ensure trusted communication in VANETs, they still have two main issues. Firstly, they do not address the other VANETs’ security and privacy requirements, including authentication (source authentication and message authentication), non-repudiation, privacy preservation, unlinkability, traceability and revocation, and resistance to common attacks [12]. To solve this issue, security, privacy, and trust can be met simultaneously by integrating an authentication scheme with a trust management approach. However, combining an authentication scheme with a trust management approach will generate a large amount of computational and communication overhead, which is another major concern in vehicular networks. Consequently, efficiency (in terms of lower computational and communication overhead) is imperative. Thus, any designed trust management scheme should meet the requirements of security, privacy, and trust while maintaining efficiency. Secondly, the current trust management approaches lack adaptability and flexibility because they are not able to detect malicious vehicles and their bogus event messages in various contexts and scenarios efficiently. There are many contexts in VANETs due to their high mobility, the random distribution of vehicles, and the presence of malicious vehicles. Existing trust management approaches are developed to work in a specific scenario (e.g., sparse scenarios [13,14] or dense scenarios [15,16,17]). The trust management approaches that are designed to operate in sparse scenarios/low density (when the network contains fewer vehicles) cannot perform efficiently when the network is dense (a network with a large number of vehicles). Other trust management approaches that are designed to operate in a dense scenario cannot operate efficiently in a sparse scenario. Therefore, a trust management scheme that can operate efficiently in various contexts/scenarios (sparse and dense scenarios) is required. Context-aware new trust management solutions are required to function in many situations (various vehicle densities, various vehicle speeds, and various densities of malicious vehicles). Making systems flexible and dynamic is the focus of the context-awareness approach. Increasing flexibility by making the most use of the available facts is the main aim of incorporating context awareness in trust management schemes [18].

To fill the gap in existing trust management approaches, this paper proposes a blockchain-assisted privacy-preserving and context-aware trust management framework to identify and revoke malicious vehicles and bogus messages in various contexts while simultaneously satisfying the efficiency, security, and privacy requirements of VANETs. The proposed framework adopts the maximum amount of available information to function in many circumstances. Additionally, blockchain technology is adopted, which can also enhance the security and privacy of the network. Blockchain is a decentralized ledger that provides a trusted environment for a secure recording of transactions in a distributed manner [19,20]. Blockchain is considered a suitable and secure data storage in VANETs because of its unique characteristics, such as decentralization, immutability, tamper resistance, privacy, availability, and transparency [19,20,21].

We provide the following contributions:

(1) First, we propose a blockchain-assisted privacy-preserving and context-aware trust management framework to identify and revoke malicious vehicles and their bogus messages from the vehicular network. The proposed framework consists of two components. The first component is a blockchain-assisted privacy-preserving authentication scheme which serves as the authentication module. The proposed authentication scheme is proposed to enable mutual authentication of vehicles and their messages and meets the security, privacy, and efficiency requirements of VANETs. The other component is a context-aware trust management scheme that consists of several modules and is used to evaluate the trustworthiness of the sender vehicles and their messages and ensure a trusted communication environment under various contexts of VANETs.
(2) Second, we adopt blockchain technology to achieve efficient and distributed authentication and revocation in VANETs. With this approach, vehicles and RSUs within VANETs can verify the authenticity of vehicles’ pseudo-identities efficiently with Proof-of-Presence (PoP) and Proof-of-Absence (PoA) mechanisms.
(3) Finally, we conduct a security analysis to illustrate that our proposed framework satisfies the trust, security, and privacy requirements of VANETs. Furthermore, we conduct an efficiency analysis to show that our authentication scheme is efficient compared to the baseline schemes in terms of low computation cost and communication overhead. Furthermore, we conduct several experiments to show that our trust management framework can identify malicious vehicles and their false/bogus messages in various contexts efficiently, compared to the baseline schemes.

The rest of this paper is structured as follows. We discuss the related works in Section 2. In Section 3, we present the proposed framework for trust management in VANETs. We present the security analysis in Section 4. The performance evaluation is discussed in Section 5. We conclude the paper in Section 6.

## 2. Related Works

Liu et al. [22] proposed a Lightweight Trust Evaluation and Privacy-Preserving (LPPTE) scheme that achieves a balance between privacy preservation and trust evaluation to facilitate the fusion of distributed data in vehicular safety applications. This scheme secures the V2V communication and satisfies authentication, privacy preservation, and efficiency in terms of computation and communication overhead. The scheme can resist false message attacks, replay attacks, and message-tampering attacks. However, this scheme does not realize the non-repudiation, unlinkability, traceability, and revocation of malicious vehicles and is not adaptive to various contexts of VANETs.

Liu et al. [23] proposed a privacy-preserving trust management (PPTM) scheme for the dissemination of emergency messages in space–air–ground integrated vehicular networks. This scheme combines strong conditional privacy preservation with trust management to secure V2V communication while having a low communication overhead. However, no details were provided on how this scheme will behave in the various contexts of VANETs. Additionally, it does not have a revocation mechanism for malicious vehicles. 

Guo et al. [24] proposed a context-aware trust management model for evaluating the trustworthiness of received messages in V2V communication. The proposed evaluation strategy adapts to context-specific scenarios through reinforcement learning (RL). The trust calculation function is a data-centric trust method constructed based on the information entropy theory in addition to the proposed RL model for determining the best strategy for the given situation by learning from the historical evaluation result. This scheme resists false message attacks. However, it is not adaptive to various contexts of VANETs and it does not realize the security and privacy requirements of VANETs, including authentication, non-repudiation, privacy preservation, unlinkability, traceability, and revocability. Gao et al. [25] proposed a trust management scheme for VANETs, which integrates direct trust and recommendation trust to identify malicious nodes in V2V communication. Historical interaction records and Bayesian inference are used to calculate the former. In the latter case, neighboring nodes are considered to calculate trust. This scheme resists false-message attacks, message-tampering attacks, message-dropping attacks, and opinion-tampering attacks. The scheme, however, lacks authentication, non-repudiation, privacy preservation, unlinkability, traceability, and revocation.

Bhargava and Verma [26] proposed an uncertainty-based Trust Model (TM) to secure V2V communication. To address uncertainty arising from information scarcity in VANETs, the Dempster–Shafer Theory (DST) is used. DST calculates a vehicle’s new trust value by combining direct and indirect trust values while considering other factors for trust calculation. This scheme can resist false-message attacks, message-dropping attacks, and message-tampering attacks. However, this scheme cannot provide authentication, non-repudiation, privacy preservation, unlinkability, traceability, and revocation.

Liu et al. [27] propose a data-oriented trust evaluation model that incorporates entity-oriented trust values into trust cascading. The proposed model allows emergency messages to be distributed by trust cascading among nearby vehicles when an emergency occurs on the road. To achieve secure V2V communication, the authenticity and trustworthiness of received messages are validated and verified using entity-centric trust values. These trust values are provided in trust certificates included in the messages. This scheme withstands false-message attacks and opinion-tampering attacks and satisfies the requirements of authentication, non-repudiation, traceability, and revocation of malicious vehicles. However, this scheme is not adaptive to various contexts of VANETs and the privacy-preservation and unlinkability requirements are not realized. 

Inedjaren et al. [28] proposed a trustworthy routing strategy based on blockchain and fuzzy logic to enhance the identification of malicious nodes in V2V communication. Their method is based on Fuzzy Logic Trusted—Optimized Link State Routing (FT-OLSR) protocol and isolates malicious vehicles using blockchain technology. This scheme resists message-dropping attacks. However, it is not adaptive to various contexts of VANETs and does not meet all the security and privacy requirements of VANETs, including authentication, non-repudiation, privacy preservation, unlinkability, traceability, and revocation.

Ghaleb et al. [29] developed an ensemble hybrid context-aware model to detect malicious vehicles. The model detects malicious vehicles sharing false mobility messages using two-hybrid and multifaceted statistical classifiers. The Hampel and Kalman filters were used to build and update a multidimensional, hybrid context-reference model. This scheme is designed to secure V2V communication by resisting false message attacks and is partially adaptive to the variations in the contexts of VANETs. However, this scheme does not meet the requirements of authentication, non-repudiation, privacy preservation, unlinkability, traceability, and revocability.

Ahmad et al. [30] proposed a novel trust evaluation and management (TEAM) framework that serves as a paradigm for designing, managing, and evaluating trust models in different contexts and with malicious vehicles in V2V communication. The proposed framework validates and evaluates a given trust model’s efficiency in the presence of malicious nodes. This framework can resist message-tampering attacks and message-delaying attacks. The framework is partially adaptive to various contexts of VANETs. This framework does not comply with the requirements for authentication, non-repudiation, privacy preservation, unlinkability, traceability, and revocation.

Ghaleb et al. [31] proposed a fuzzy-logic-based scheme for context-aware misbehavior detection in V2V communication. Fuzzy variables represent the vehicle’s context and behavior. The vehicles’ context and behavior are evaluated through a fuzzy inference system. This scheme is designed to secure V2V communication. It resists malicious vehicle attacks and is partially adaptive to the various contexts of VANETs. The authentication, non-repudiation, privacy preservation, unlinkability, traceability, and revocation requirements are not met by this scheme.

Rehman et al. [32] proposed a framework based on a context-aware cognitive approach to secure V2V communication. Based on the received message, the framework creates a context for an event by cognitively learning the environment. Furthermore, this framework provides a method for detecting malicious nodes based on anomalous outliers. This scheme resists malicious vehicles and is partially adaptive to various contexts of VANETs. However, this scheme does not meet the VANET requirements of authentication, non-repudiation, privacy preservation, unlinkability, traceability, and revocability. 

To guarantee the trustworthiness of received messages, Ghajar et al. [33] provide a Bayesian formula for trust management and some blockchain-based data. Vehicles verify the accuracy of the messages they have received and calculate the trust values of the sender vehicles. RSUs receive the computed trust values from vehicles. Each RSU generates a block that includes the trust values via a sharding consensus mechanism. This scheme provides efficient storage for vehicles’ trust values. However, this scheme is not context-aware and does not meet the authentication, non-repudiation, privacy preservation, unlinkability, traceability, and revocability requirements of VANETs.

A Bayesian trust inference model is presented by Chukwuocha et al. [34] that takes into account both the message’s trustworthiness and the vehicles’ trustworthiness. The model computes the beta distribution using real-time event data messages. Additionally, the authors divide the road network into zones to decrease communication overhead and improve scalability. RSUs are included in every zone. The RSUs work together to create a blockchain network. The vehicles transmit to the RSU the computed trust values to store them in the blockchain. This scheme is designed to secure V2V communication by identifying false message attacks. The security and privacy requirements are not all met, and context awareness is not provided.

Hasrouny et al. [35] presented a trust management and revocation approach based on the behavior of groups of participating vehicles. Misbehavior detection mechanisms are used to exclude malicious entities. This scheme satisfies the authentication, non-repudiation, privacy preservation, traceability, and revocability requirements in VANETs. However, unlinkability and context awareness are not provided by this scheme.

Based on the above analysis, the above schemes focus on securing V2V communication alone (without considering V2I communication) and no scheme can achieve security, privacy, trust, and efficiency simultaneously. Additionally, the previous schemes are either designed to operate in a specific scenario or are partially context-aware. Our proposed framework is context-aware and satisfies simultaneously all the aspects of security, privacy, trust, and efficiency (in terms of low computational and communication overhead) for both the V2V and V2I communications. 

A comparison of the proposed trust framework and other approaches is provided in Table 1.

## 3. Proposed Trust Management Framework

### 3.1. Preliminaries

Authentication is necessary for ensuring the security of a network. Authentication is created at the node level and message level. Authentication at the node level enables the receiver node to verify that the sender node is registered and legitimate and ensures that the received message originates from a legitimate source. Authentication at the message level ensures the received message’s integrity (that the message has not been modified during transmission). Without a security service, malicious attackers can alter the messages sent by network nodes. Additionally, without identity privacy preservation, the identity privacy of the network nodes can be disclosed and malicious entities/attackers may use this information to perform tracking attacks.

Trust is of paramount importance in VANETs. Although authentication ensures that messages are authenticated and originate from authenticated vehicles, authenticated vehicles may behave maliciously and send bogus yet authenticated messages. Trust management approaches are used to detect and eliminate these bogus messages and the malicious vehicles generating them. However, due to the diverse contexts of VANETs (variations in the network vehicles’ density and mobility), an effective trust management approach needs to be context-aware to operate and adapt effectively to these contexts. 

Context awareness is the basis of our proposed framework for evaluating trust in VANETs. Context awareness is primarily used to increase the flexibility of the proposed framework by leveraging the maximum available data so that it can operate efficiently in various contexts. 

### 3.2. Adversary Model

A malicious vehicle is considered the adversary. Many malicious vehicles could be present on a network. A malicious vehicle can launch combined attacks by alternating its malicious behavior and executing any of the following attacks:

- False message attack: False or bogus messages are generated by malicious vehicles to mislead other vehicles or RSUs. These attacks may lead honest drivers to take incorrect actions, such as decelerating, braking, or considering alternate routes. A malicious vehicle may engage in bogus message attacks through collusion (collaborating with other malicious vehicles to carry out the attacks together), non-collusion (carrying out the attack individually), or by on–off patterns (alternating its behavior by sending false messages for some time before switching to sending messages with correct information and vice versa).
- Opinion-alteration attack: An attacker can propagate false trust opinions about other vehicles to make them appear benign or legitimate so the malicious vehicles remain undetected. This attack attempts to disrupt trust evaluation, making it more difficult to identify malicious attackers [36].

### 3.3. Details of the Proposed Trust Framework

**Figure 1:**
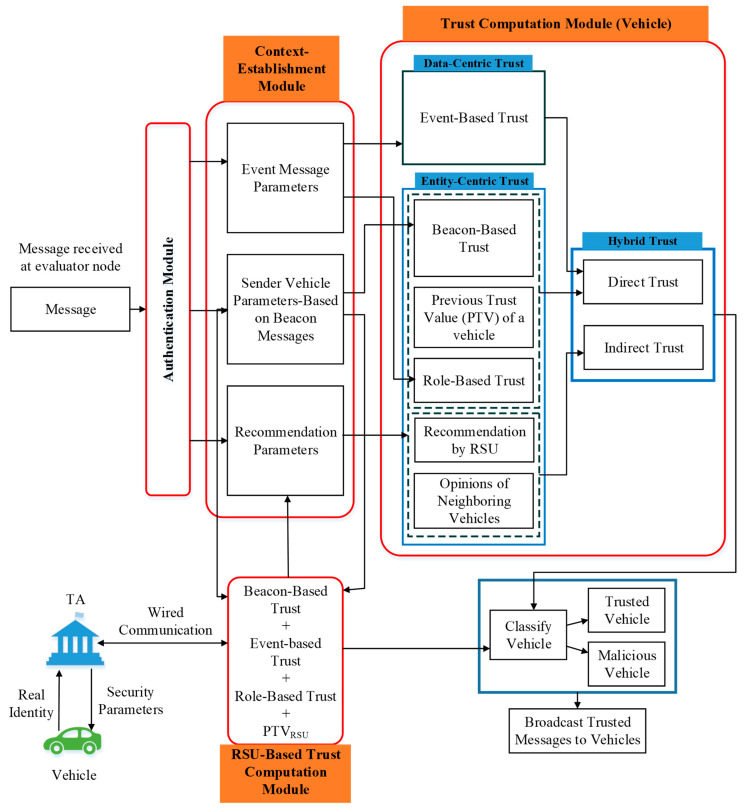
Proposed trust management framework.

This section details our trust management framework for VANETs. Figure 1 shows the proposed trust management framework. It is composed of two components: a blockchain-assisted privacy-preserving authentication scheme and a context-aware trust management scheme. The authentication module is composed of the blockchain-assisted privacy-preserving authentication scheme. The other modules are the context-aware trust management scheme, which includes the context establishment module (parameters extraction), the trust computation module on the vehicle, the trust evaluation module on the RSU, and the decision module. The network participants include the trusted authority (TA), RSUs, and vehicles. 

TA: The TA is primarily in charge of initializing the parameters for registered vehicles and RSUs. The TA also generates a partial private key and a pseudonym for each vehicle. The TA can trace and revoke malicious vehicles when it receives a warrant from the RSU. Through the pseudonym of the vehicle, the TA can identify the vehicle’s real identity. Furthermore, the TA stores the generated pseudo-identities of registered vehicles in the valid pseudo-identities blockchain and the revoked pseudo-identities of malicious vehicles in the revoked pseudo-identities blockchain. The TA distributes the latest versions of both blockchains to the RSUs.

RSUs: RSUs are situated on both sides of the road. V2I communication mode is used for communications between RSUs and vehicles. RSUs receive beacon messages and traffic event information from the vehicles in their vicinity. Additionally, RSUs verify the authenticity and trustworthiness of the messages received from vehicles. RSUs also store the trust values of vehicles in their databases. The RSU utilizes the two blockchains to verify the legitimacy of the sender vehicle’s pseudo-identity during authentication. The latest blockchains (received from the TA) are also broadcast by RSUs to vehicles in their communication range. Event notifications are also broadcast by RSUs to the vehicles. Secure wired channels are used by RSUs to communicate with the TA. RSUs send lists of malicious vehicles to the TA for their revocation from the vehicular network.

Vehicles: When a road event is detected, vehicles send information about it to neighboring vehicles and RSUs. Vehicles can also inquire about the trust values of other vehicles from the RSUs. Vehicles can communicate with other neighboring vehicles and adjacent RSUs using the V2V and V2I communication modes, respectively. Additionally, vehicles can verify the authenticity and trustworthiness of received messages before acting upon them. The two blockchains are utilized by the receiver/evaluator vehicle to verify the legitimacy of a sender vehicle’s pseudo-identity during authentication. The receiver/evaluator vehicle calculates the trust value of the sender vehicles and stores these trust values in its trust database. 

Trust database: A database is also maintained by every vehicle and RSU to keep track of the trust ratings of all the other sender vehicles they interact with. With the help of a database, receiver nodes (vehicles/RSUs) can check for the previous trust values of sender vehicles (previously interacted vehicles) during trust computations. The receiver node includes any previous trust value (from its local database) along with the available trust information to calculate the new trust value of the vehicle. If there is no previous trust value (i.e., newly interacting vehicle), the receiver node considers the initial trust value and creates a new record for the sender vehicle in the database. Therefore, the database will help the receiver node to retrieve, compute, update, and store the trust values of the sender vehicles locally. VANETs have high mobility and a rapidly changing topology, and they exchange time-sensitive and life-critical information. The use of a database has been demonstrated to be efficient, as it allows the receiver node to make trust computations, identify, and eliminate malicious vehicles at the node level, making the network more secure and time-efficient. Furthermore, the receiver nodes store in their local databases only the pseudo-identities and the computed trust values of the sender vehicles they interact with.

Valid Pseudo-Identities Blockchain (VPID-BC): All unrevoked vehicle pseudo-identities produced by TA are stored in the VPID-BC public database. The VPID-BC enables the evaluator/receiver node (vehicle/RSU) to conduct the sender vehicle’s pseudo-identity proof-of-presence (PoP) efficiently with O(logN) time, where N stands for the Merkle tree’s total number of leaves.

Revoked Pseudo-Identities Blockchain (RPID-BC): The TA revokes all pseudo-identities of malicious vehicles based on the revocation lists sent by the RSUs. The revoked pseudo-identities are recorded in the RPID-BC blockchain. The sender vehicle’s pseudo-identity proof-of-absence (PoA) can be performed by the evaluator/receiver node (vehicle/RSU) efficiently with O(logN) time.

In VPID-BC, chronological Merkle trees (CMTs) are used to record the generated pseudo-identities. CMTs and lexicographical Merkle trees (LMTs) are both included in RPID-BC. The root of the LMT is updated to reflect the inclusion of new revoked pseudo-identities whenever they are added. CMTs store pseudo-identity revocation transactions and LMT roots chronologically. The LMT pseudo-identity root and the CMT transaction root are both included in block headers.

Every vehicle and RSU is equipped with the proposed framework, enabling the receiver node (vehicle/RSU) to identify malicious vehicles and the bogus messages they generate and eliminate them from the network in various contexts of VANETs.

When a receiver/evaluator node (vehicle/RSU) receives a critical event alert from a neighboring vehicle, the proposed framework enables the receiver/evaluator node (vehicle/RSU) to anonymously authenticate and evaluate the trustworthiness of the sender vehicle and its message. The proposed framework authenticates the sender vehicle and its message using the authentication module. Next, the proposed trust management framework establishes a context for the event by making use of several parameters and performs a trustworthiness evaluation of the sender vehicle and its message. The proposed framework performs the trust evaluation and calculates the trust value of the sender vehicle by integrating direct trust and indirect trust. Once the trust value of the sender vehicle is computed, the receiver node (vehicle/RSU) will compare it with a pre-defined threshold. If the trust value of the sender vehicle is greater than the pre-defined threshold, the receiver/evaluator node (vehicle/RSU) will accept and act upon the received message from the sender vehicle. Otherwise, the received message will be discarded. Next, the sender’s trust value will be updated and stored in the receiver/evaluator node (vehicle/RSU) local database. Vehicles with a trust value less than the predefined threshold are considered malicious. If these vehicles continue to send false/bogus messages, the receiver/evaluator node puts them (their pseudo-identities) on the revocation list and sends it to the TA to revoke them (their pseudo-identities) and add the revoked pseudo-identities of malicious vehicles to the revocation blockchain. The revoked malicious vehicles will not be able to participate in the vehicular network. 

#### 3.3.1. A Blockchain-Assisted Privacy-Preserving Authentication Scheme

The first component of the proposed framework is a blockchain-assisted privacy-preserving authentication scheme which serves as the authentication module. This module is in charge of the early detection of malicious vehicles and bogus event messages in the network. This module performs a privacy-preserving mutual authentication between the sender vehicle and the receiver node (vehicle/RSU). Additionally, it enables the receiver node to check the authenticity and integrity of received messages. The message received at the evaluator node (Vehicle/RSU) is only valid once its authenticity and integrity have been confirmed.

The authentication module contains the following steps: Setup, Generation of Pseudonym, Generation of Vehicle Key, Generation of RSU Key, Generation of Signature, Verification of Signature, Generation of Aggregate Signature, and Verification of Aggregate Signature. The notations used in the proposed authentication scheme are listed in Table 2.

1. Setup
  - Firstly, the TA chooses p and q as two secure prime numbers, then selects $a,b\in Z_{q}^{*}$, and generates an elliptic curve E based on the equation $E:y^{2}=x^{3}+ax+b\;mod\;p$, where 4$a^{3}$ + 27$b^{2}$ (mod *p*) ≠ 0 and point P consists of all the points on E, to generate the cyclic group G.
  - The AT randomly selects $s\in Z_{q}^{*}$ as its secret master key and sets the public key as ${TA}_{pub}=s\cdot P$. The secret master key is kept securely in its repository.
  - The TA randomly selects ${\rho \in Z}_{q}^{*}$ and computes R=ρ·P.
  - The TA chooses cryptographic hash functions: $h1:{\left\{0,1\right\}}^{*}\to Z_{q}^{*},h2:{\left\{0,1\right\}}^{*}\times G\to Z_{q}^{*},h3:{\left\{0,1\right\}}^{*}\times G\times G\to Z_{q}^{*},and\;h4:{\left\{0,1\right\}}^{*}\times G\times G\times G\to Z_{q}^{*}$.
  - Finally, the TA publishes: $\left\{h1,h2,h3,h4,p,q,a,b,P,{TA}_{pub},R\right\}$ as the system parameters.
2. Generation of Pseudonym
  Through a secure channel, the vehicle provides the TA with its real identity ${RID}_{i}$ as received from the manufacturer (MVM).
  The TA randomly chooses $r_{i}\in Z_{q}^{*}$ and generates a pseudonym as below:
  The TA computes ${SID}_{i}={RID}_{i}⊕r_{i}\cdot {TA}_{pub}$.
  - The TA computes ${VID}_{i}={RID}_{i}⊕h_{1}\left(s\cdot {SID}_{i}\right)$.
  - Then, the TA sends the generated pseudonym ${PID}_{i}=({VID}_{i},T_{i})$ to the vehicle $V_{i}$.
  - The TA stores $\left\{{PID}_{i},{SID}_{i},r_{i}\right\}$ in its database.
  - The TA can retrieve the vehicle $V_{i}$’s real identity by computing ${RID}_{i}={SID}_{i}⨁r_{i}{TA}_{pub}$.
3. Generation of Partial Private Key
  - The TA randomly chooses $u_{i}\in Z_{q}^{*}$.
  - The TA computes $U_{i}=u_{i}\cdot P$, $\vartheta_{i}=h2({PID}_{i},R)$ and generates the vehicles’ partial private key as: ${ppk}_{i}=u_{i}+\vartheta_{i}\times \rho (mod\;p)$.
  - Finally, the TA sends {$U_{i},{ppk}_{i}\}$ to the vehicle $V_{i}$.
4. Generation of Vehicle Key
  - As soon as the partial private key ${ppk}_{i}$ is received, the vehicle $V_{i}$ calculates $\vartheta_{i}=h2({PID}_{i},R)$ and checks whether ${ppk}_{i}\cdot P=U_{i}+\vartheta_{i}\cdot R$. If this condition is met, the partial private key ${ppk}_{i}$ is valid.
  - The vehicle randomly chooses two numbers, $\beta_{i},\gamma_{i}\in Z_{q}^{*}$, and calculates $f_{i}=h3({PID}_{i})$, ${vsk}_{i}=\beta_{i}\cdot f_{i}$, $K_{i}=\gamma_{i}\cdot P$, $Q_{i}={vsk}_{i}\cdot P$.
  - The vehicle sets its full private key as ${SK}_{i}=({ppk}_{i},{vsk}_{i})$, and its public key as ${PK}_{i}=Q_{i}+U_{i}$.
5. Generation of RSU Key
  The TA generates the ${RSU}_{i}$’s public key ${PU}_{i}$ and private key ${PR}_{i}$ by randomly selecting $a_{i}\in Z_{q}^{*}$, and assigning the private key as ${PR}_{i}=a_{i}$, then calculates the public key as ${PU}_{i}$= $a_{i}\cdot P$.
6. Generation of Signature
  To ensure message integrity, the vehicle needs to generate its signature on the message before sending it to another vehicle or RSU. To do so, it executes the following steps:
  - The vehicle $V_{i}$ randomly chooses a number $l_{i}\in Z_{q}^{*}$ and calculates $L_{i}=l_{i}\cdot K_{i}$.
  - $V_{i}$ computes $n_{i}=h4({PID}_{i},{PK}_{i},L_{i},M_{i},t_{i})$, where $t_{i}$ is the timestamp, and calculates $D_{i}=l_{i}\cdot \gamma_{i}+n_{i}\left({vsk}_{i}+{ppk}_{i}\right)(mod\;q)$.
  - Then, the vehicle sets the signature $\sigma_{i}=(L_{i},D_{i})$ on message $M_{i}$. Finally, the vehicle $V_{i}$ sends ${\{PID}_{i},{PK}_{i},M_{i},\sigma_{i},t_{i}\}$ to the receiver/evaluator node for verification.
7. Verification of Signature
  When a message tuple ${\{PID}_{i},{PK}_{i},M_{i},\sigma_{i},t_{i}\}$ is received, the receiver/evaluator node (vehicle/RSU) checks the signature on the message $M_{i}$ which has been signed by the sender’s vehicle $V_{i}$. The receiver/evaluator node will execute the following steps:
  - The receiver/evaluator node (vehicle/RSU) checks whether the timestamps $T_{i}$ and $t_{i}$ in the pseudonym ${PID}_{i}$ and in the received message ${\{PID}_{i},{PK}_{i},M_{i},\sigma_{i},t_{i}\}$, respectively, are within acceptable time ranges before validating the message $M_{i}$. If one of them is invalid, the receiver/evaluator node aborts; otherwise, proceed to the next step.
  - The receiver/evaluator node checks the received ${PID}_{i}$ against VPID-BC and RPID-BC to be sure of its presence in VPID-BC and absence in RPID-BC. In other words, ${PID}_{i}$ has been allocated to the vehicle $V_{i}$ and has not been revoked by TA. The PoP and PoA of the ${PID}_{i}$ in VPID-BC and RPID-BC are performed efficiently with O(logN) time.
  - If the ${PID}_{i}$ is not revoked, the receiver/evaluator node can carry on with the verification.
  - The receiver/evaluator node checks the validity and accuracy of the message $M_{i}$ generated by vehicle $V_{i}$ by executing the following steps:
  - The receiver/evaluator node calculates $n_{i}=h4({PID}_{i},{PK}_{i},L_{i},M_{i},t_{i})$ and $\vartheta_{i}=h2({PID}_{i},R)$.
  - The receiver/evaluator node checks whether $D_{i}\cdot P=L_{i}+n_{i}\cdot ({PK}_{i}+\vartheta_{i}\cdot R)$. If the equation holds, the message will be accepted. Otherwise, reject.
  Proof of Correctness:
  $$\begin{array}{cc} D_{i}\cdot P & =\left(l_{i}\cdot \gamma_{i}+n_{i}\left({vsk}_{i}+{ppk}_{i}\right)\right)\cdot P\\ & =\left(l_{i}\cdot \gamma_{i}+n_{i}\left({vsk}_{i}+u_{i}+\vartheta_{i}\times \rho \right)\right)\cdot P\\ & =l_{i}\cdot \gamma_{i}\cdot P+n_{i}\left({vsk}_{i}+u_{i}+\vartheta_{i}\times \rho \right)\cdot P\\ & =l_{i}\cdot K_{i}+n_{i}\cdot \left({vsk}_{i}\cdot P+u_{i}\cdot P+\vartheta_{i}\cdot \rho \cdot P\right)\\ & =L_{i}+n_{i}\cdot \left(Q_{i}+U_{i}+\vartheta_{i}\cdot R\right)\\ & =L_{i}+n_{i}\cdot \left({PK}_{i}+\vartheta_{i}\cdot R\right) \end{array}$$
8. Generation of Aggregate Signature
  When a large number of messages ${\{PID}_{i},{PK}_{i},M_{i},\sigma_{i},t_{i}\}$ are received from different vehicles $V_{i}(i=1,2,\cdots ,n)$, the RSU computes $L=\sum_{i=1}^{n}L_{i}$ and $D=\sum_{i=1}^{n}D_{i}$ and outputs the aggregate signature σ=(L,D). Multiple signatures are consolidated into one short signature σ by the RSU. In this way, the computation cost and communication overhead can be reduced.
9. Verification of Aggregate Signature

The RSU performs the following calculation to verify the validity of the aggregate σ signature.

$$D\cdot P=L+\sum_{i=1}^{n}\left(n_{i}\cdot ({PK}_{i}+\vartheta_{i}\cdot R)\right)$$

If the equation mentioned above holds, the RSU will accept σ; otherwise, it is rejected.

Proof of Correctness:

$$\begin{array}{c} D\cdot P=\sum_{i=1}^{n}D_{i}P\\ =\sum_{i=1}^{n}\left(l_{i}\cdot \gamma_{i}+n_{i}\left({vsk}_{i}+{ppk}_{i}\right)\right)\cdot P\\ =\sum_{i=1}^{n}\left(l_{i}\cdot \gamma_{i}+n_{i}\left({vsk}_{i}+u_{i}+\vartheta_{i}\times \rho \right)\right)\cdot P\\ =\sum_{i=1}^{n}\left(l_{i}\cdot \gamma_{i}\cdot P+n_{i}\left({vsk}_{i}+u_{i}+\vartheta_{i}\times \rho \right)\cdot P\right)\\ =\sum_{i=1}^{n}\left(l_{i}\cdot K_{i}+n_{i}\cdot \left({vsk}_{i}\cdot P+u_{i}\cdot P+\vartheta_{i}\cdot \rho \cdot P\right)\right)\\ =\sum_{i=1}^{n}\left(L_{i}+n_{i}\cdot \left(Q_{i}+U_{i}+\vartheta_{i}\cdot R\right)\right)\\ =\sum_{i=1}^{n}\left(L_{i}+n_{i}\cdot \left({PK}_{i}+\vartheta_{i}\cdot R\right)\right)\\ =L+\sum_{i=1}^{n}\left(n_{i}\cdot \left({PK}_{i}+\vartheta_{i}\cdot R\right)\right) \end{array}$$

##### Communication Scenario

**Figure 2:**
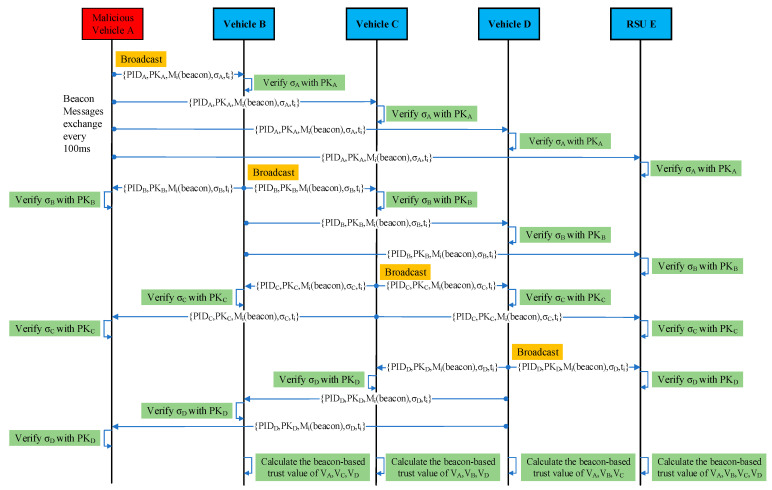
Communications between the RSU and vehicles during beacon messages exchange.

Vehicles periodically send beacon messages to share information about their mobility status with their one-hop neighboring vehicles and the nearest RSU. A vehicle’s mobility status includes its speed, location, and direction, which helps keep vehicles aware of their surroundings to improve traffic safety and efficiency. The vehicles also generate and broadcast another type of message (an event message) when they encounter an event on the road (e.g., traffic accident, traffic jam, etc.) to inform the other vehicles about it. Figure 2 shows that vehicles V_A_, V_B_, V_C,_ and V_D_ periodically share beacon messages (containing their driving status: position, speed, and direction) with each other and with the nearest roadside unit RSU_E_. Every vehicle (V_A_, V_B_, V_C,_ and V_D_) and RSU_E_ authenticates and evaluates the trustworthiness of the sender vehicles based on the received beacon messages (beacon-based trust). 

**Figure 3:**
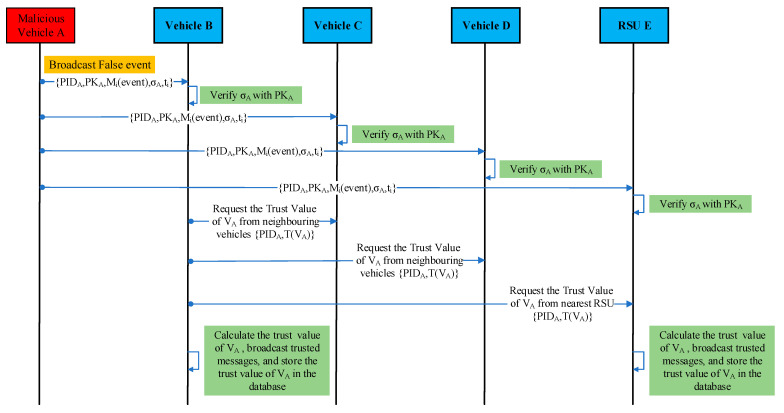
Communications between the RSU and vehicles during event message exchange and trust calculation.

In this scenario, vehicles V_A_, V_B_, V_C,_ and V_D_ are moving on the road within the same communication range. A malicious vehicle V_A_ suddenly broadcasts a false safety event message claiming that there is traffic congestion ahead on the road. The neighboring vehicles, V_B_, V_C_, V_D_, and the nearest RSU_E_, receive this alert (event message). Vehicles V_B_, V_C_, V_D_, and RSU_E_ verify the authenticity and trustworthiness of the alert before acting upon it. However, the alert generated by vehicle V_A_ is false/bogus. The receivers V_B_, V_C_, V_D_, and the nearest RSU_E_ evaluate the trustworthiness of the sender vehicle V_A_ and its event message and find that the event message is a false/bogus message, classify the sender vehicle V_A_ as a malicious vehicle, and discard the received event message. Figure 2 and Figure 3 illustrate the communications between the RSU and vehicles. In Figure 3, vehicles V_C_ and V_D_ also perform the same steps as vehicle V_B_ for trust calculation. A representation of data in beacon messages $M_{1}(beacon)$ sent by vehicles is shown in Table 3. Table 4 illustrates the contents of the event message $M_{1}(event)$ sent by vehicle V_A_ to V_B_, V_C_, V_D_, and RSU_E_.

#### 3.3.2. A Context-Aware Trust Management Scheme

The second component of the proposed framework is a context-aware trust management scheme. This scheme is composed of several modules: Context Establishment/Parameters Extraction Module, Trust Computation Module on Vehicle, Trust Calculation Module on the RSU, and Decision Module.

##### Context Establishment/Parameter Extraction Module

This module performs parameter extraction, which follows the authentication process as shown in Figure 1. To obtain parameters, the proposed framework on the receiver node (vehicle/RSU) obtains information about the sender vehicle status from the authenticated beacon messages (parameters from authenticated beacon messages). Additionally, information about an event is filtered out from the authenticated event messages that were received (parameters from the authenticated event messages). Furthermore, information obtained from nearby RSUs and neighboring vehicles is also considered. 

##### Trust Computation Module on Vehicle

After obtaining the necessary information from the previous module, the trust computation module enables the receiver/evaluator vehicle to evaluate the trustworthiness of the sender vehicle and its messages. The trust computation module consists of entity-centric, data-centric, and hybrid trust sub-modules. The output of the entity-centric and data-centric sub-modules makes the input of the hybrid trust the sub-module. The hybrid trust sub-module is based on direct trust and indirect trust computations. The direct trust computation is performed using data-centric parameters (event-based trust) and some entity-centric parameters (role-based trust, beacon-based trust, previous trust value). The indirect trust computation is performed using the remaining entity-centric parameters (recommendations by RSU and opinions of neighboring vehicles). The output of the hybrid trust sub-module is considered the output of the trust computation module on the vehicle. The output of this module is the final trust value of the sender vehicle. 

The entity-centric, data-centric, and hybrid trust sub-modules are described below.

*A.*  *Entity-Centric Trust*

This sub-module computes the trust value of the sender vehicle by integrating various trust computation techniques: (1) role-based trust (RBT), (2) beacon-based trust, (3) opinions of neighboring vehicles, (4) recommendations from RSUs, and (5) the sender vehicle’s previous trust value (PTV). 

Role-based Trust (RBT): In RBT, trust is incorporated from vehicles that are highly trusted in the network and are approved by higher authorities. These vehicles transmit messages that are most trusted. The RBT is considered an additional factor when computing the trust of the sender vehicle. There are three different kinds of vehicles ($V_{Role}$) in our framework:

(1) Authority vehicles (AV) (such as traffic patrols)—These vehicles are approved by a centralized authority or specific department, and the messages they transmit are highly trusted.
(2) Public services (PS) vehicles (such as buses, road upkeep vehicles, engineering vehicles, sanitation trucks, etc.)—As they have been authorized by specific departments, they are highly trusted.
(3) Ordinary vehicles (OV) (such as private cars, taxis, freight vehicles, etc.)—Individuals primarily control these vehicles.

To ensure realistic behavior, we consider that AV vehicles are mostly trusted vehicles in the network. Additionally, PS disseminates trusted information because it has been approved by a specific department. For OV vehicles, vehicle authorization occurs at the vehicle level. Consequently, RBT can be modeled using the equation below.

(1) $$RBT(V_{i})=\left\{\begin{array}{c} 1\\ 0.8\\ 0.3 \end{array}\right.\begin{array}{c} if\;V_{Role}=AV\\ if\;V_{Role}=PS\\ if\;V_{Role}=OV \end{array}$$

The $V_{Role}$ attribute of the vehicle is included in the event message. 

The VANET is a large-scale network, so we expect that a majority of vehicles will be ordinary and only a minority will be AV or PS. The equation demonstrates that messages sent by the first two types of vehicles can be highly trusted. 

Beacon-based Trust: The trust calculated based on the cosine similarity between beacon messages received from the sender vehicle.

Sending beacon messages: Single-hop beacon messages about the vehicle’s driving status (position, velocity, and direction) are broadcast periodically by vehicles’ OBUs to their neighboring vehicles on the road to keep them aware of their surroundings and to improve traffic safety and efficiency.

The structure of the beacon message, $M_{i}(beacon)$, is as follows:

$$M_{i}=<\left(x_{i},y_{i}\right),v_{i},d_{i}>$$

A vehicle broadcasts this message to all its neighboring vehicles, notifying them of its location $\left(x_{i},y_{i}\right)$, velocity ($v_{i}$), and direction ($d_{i}$). The location $\left(x_{i},y_{i}\right)$ also refers to the latitude and longitude of the source (sender) of the beacon message and is represented by Lat and Long or $\left(Lat,Long\right)$. The beacon message $M_{i}$ is signed by each vehicle’s private key ${SK}_{i}$ to generate the signature $\sigma_{i}$.

Afterward, the vehicle broadcasts the message tuple ${\{PID}_{i},{PK}_{i},M_{i},\sigma_{i},t_{i}\}$.

Receiving beacon messages: When the receiver/evaluator node EV (vehicle/RSU) receives the beacon messages from the sender vehicle, it checks if the timestamp $t_{i}$ of the beacon message is fresh or not. The time of generating the beacon message is referred to as the timestamp $t_{s}$ which corresponds to the reporting time.

The evaluator node EV evaluates the sender vehicles’ trustworthiness based on the received beacon messages by comparing the claimed values (position, velocity, and direction) received in the most recent beacon (claimed values) to the estimated values (from the previously received beacon messages). The estimated vector and the claimed vector, which comprise a set of data representing vehicle position, velocity, and direction, are compared using cosine similarity to determine the angle between them. Cosine similarity is defined as follows:

(2) $$Cos\_sim(A,B)=\frac{A\cdot B}{\left\|A\right\|\left\|B\right\|}$$

(3) $$Cos\_sim(A,B)=\frac{\sum_{i=1}^{n}A_{i}B_{i}}{\sqrt{\sum_{i=1}^{n}A_{i}^{2}}\sqrt{\sum_{i=1}^{n}B_{i}^{2}}}$$

where the estimated vector $A_{i}$ represents the estimate computed from previously received beacons, and $B_{i}$ represents the claimed vector values extracted from the most recent beacon. Both of these vectors are composed of four elements: (x, y, v, and d), where (x, y) denotes the position, v is the vehicle’s velocity, and d is the vehicle’s direction. We utilize Equation (4) to evaluate the trustworthiness of the beacon messages of encountered vehicles. For all beacon messages received in time t:

(4) $$T_{beacon-based}(EV,V_{j})=\frac{\sum_{i=1}^{n}Cos\_Sim(A_{i},B_{i})}{b}$$

A receiver/evaluator node EV calculates the adjacent vehicle’s $V_{j}$ beacon trustworthiness $T_{beacon-based}(EV,V_{j})$ by using Equation (4), where *b* indicates the number of beacons to be considered.

Opinion of neighboring vehicles: The evaluator/receiver node EV requests the trust value of the sender vehicle $V_{j}$ from the neighboring vehicles $V_{x}$. The average trust value that the neighboring vehicles $V_{x}$ have about the vehicle $V_{j}$ is indicated by $T_{onv}$ and is calculated as follows:

(5) $$T_{onv}=\frac{\sum_{x=1}^{N}V_{x}(V_{j})}{N}$$

where *N* represents the number of neighboring vehicles.

Recommendation by RSU: The receiver/evaluator vehicle queries the sender vehicle’s trust value from the adjacent RSU. The RSU retrieves the trust value of this vehicle from its database and sends it to the requester vehicle. This entity-centric parameter is used as input to the hybrid-trust sub-module.

The sender vehicle’s previous trust value (PTV): If the receiver/evaluator vehicle has past interactions with the sender vehicle, then there will be a previous trust value for the sender vehicle in its database. The receiver/evaluator vehicle retrieves the previous trust value of the sender vehicle from its local database and includes it in the trust computation. This entity-centric parameter is also used as input to the hybrid-trust sub-module.

*B.*  *Data-Centric Trust/Event-Based Trust*

Event-Based Trust is calculated to check the trustworthiness of event messages received from the sender vehicles. The Euclidean distance between received event messages is used to calculate the data-centric trust/Event-Based Trust. The sender vehicle $V_{1}$ broadcasts an event message ${\{PID}_{i},{PK}_{i},M_{i},\sigma_{i},t_{i}\}$ in the time $t_{i}$ to report an event $E_{1}$ in location $P_{1}$. The event message will be received by other vehicles (such as $V_{2}$, $V_{3}$, and $V_{4}$) in the communication range of $V_{1}$. Several vehicles may also have sensed the event and broadcast it to the other vehicles. To simplify the description, some objects are formally defined as follows. *M* = {$M_{1}$, $M_{2}$, …, $M_{i}$} is the collection of received event messages. *V* = {$V_{1}$, $V_{2}$, …, $V_{i}$} is the set of vehicles sending the event messages about the same event.

The event message $M_{i}$(event) received by the evaluator node EV from another vehicle is represented as follows:

$$M_{i}\left(event\right)=<V_{Role},E_{Type},(E_{x},E_{y}),(L_{x},L_{y})>$$

A vehicle with the ${PID}_{i}$ sends an event message $M_{i}\left(event\right)$. $V_{Role}$ indicates the vehicle’s role, $E_{Type}$ represents the event type where $E_{Type}\in${Traffic accident, Traffic jam, Ice on the road, Road construction}, $\left(E_{x},E_{y}\right)$ is the location of the event, $\left(L_{x},L_{y}\right)$ is the sender vehicle’s location when generating the event message, and $t_{i}$ is the reporting time. When vehicles detect an event, the type of event, the event location, and the vehicles’ locations are typically similar. The evaluator/receiver node EV calculates the received messages’ trust value by computing the Euclidean distance between them. The Euclidean distance is used to compute the similarity between data included in the event messages $\left\{E_{Type},\left(E_{x},E_{y}\right),(L_{x},L_{y})\right\}$. The similarity between two messages decreases as their distance increases. The similarity between two messages received from two vehicles based on Euclidean distance is calculated using Equation (6):

(6) $$ECD=\frac{\sum_{i=1}^{n}{\left(X_{i}-Y_{i}\right)}^{2}}{n}$$

In Equation (6), *X* and *Y* are vectors. $X_{i}$(or $Y_{i}$) represents the value of the *i*-dimension in the vector, and n is the number of dimensions in the vector.

If there are m event messages received from m neighboring vehicles, the average Euclidean distance (AD) between them is computed by Equation (7):

(7) $$AD=\frac{ECD(between\;all\;the\;messages)}{m}$$

The trust value based on the Euclidean distance between the received event messages is calculated by the evaluator/receiver node EV using Equation (8):

(8) $$T_{Event-based}\left(EV,V_{j}\right)=\frac{1}{1+AD}$$

where $V_{j}$ is the sender vehicle.

*C.*  *Hybrid Trust/Combined Trust*

The input of the hybrid trust sub-module is the output of the entity-centric and data-centric trust sub-modules. Therefore, based on entity-centric and data-centric trust parameters, direct and indirect trust are computed. Next, the direct and indirect trust computations are combined to calculate the hybrid trust. 

The direct trust DT is calculated as follows:

(9) $$DT=\frac{T_{beacon-based}(EV,V_{j})+T_{Event-based}\left(EV,V_{j}\right)+RBT(V_{j})+PTV(V_{j})}{4}$$

The indirect trust IDT is calculated as follows:

(10) $$IDT=\frac{Rec(RSU,V_{j})+T_{onv}}{2}$$

where $Rec(RSU,V_{j})$ indicates the trust value that the nearby RSU has about the vehicle $V_{j}$ in its database.

The hybrid trust $HT(EV,V_{j})$ which indicates the final trust value of the sender vehicle $V_{j}$ as computed by the receiver/evaluator vehicle EV is calculated as follows:

(11) $$HT(EV,V_{j})=\alpha \cdot DT(EV,V_{j})+(1-\alpha )\cdot IDT(EV,V_{j})$$

where 0<α<1.

##### Trust Calculation Module on the RSU

The receiver/evaluator (EV) RSU computes the sender vehicle’s final trust value using the following equation:

(12) $$HT\left(EV,V_{j}\right)=\frac{T_{beacon-based}\left(EV,V_{j}\right)+T_{Event-based}\left(EV,V_{j}\right)+RBT(V_{j})+PTV(V_{j})}{4}$$

where $V_{j}$ is the sender vehicle. $HT\left(EV,V_{j}\right)$ is the final trust value of the sender vehicle $V_{j}$, $T_{beacon-based}\left(EV,V_{j}\right)$ is calculated by the receiver/evaluator RSU (EV) based on the beacon messages received from $V_{j}$ using Equations (3) and (4), and $T_{Event-based}\left(EV,V_{j}\right)$ is determined based on the Euclidean distance between the received event messages from the sender vehicles using Equations (6)–(8). $RBT\left(V_{j}\right)$ is calculated based on Equation (1). $PTV(V_{j})$ is the previous trust value of the sender vehicle $V_{j}$ retrieved from the RSU database. 

##### Decision Module

The input of the decision module is the output of the trust computation module on the vehicle/RSU, which is the final trust value of the sender vehicle. The decision module enables the receiver/evaluator node EV (vehicle/RSU) to classify the sender vehicle as a trusted vehicle or malicious vehicle. A vehicle’s trust value is estimated between 0 and 1, with maximum trust being represented by 1, trusted represented as more than or equal to the trust threshold $T_{thr}$, and untrusted represented as 0 or less than the trust threshold $T_{thr}$.

(13) $$Decision=\left\{\begin{array}{c} Trusted\;Vehicle\\ Malicious\;Vehicle \end{array}\right.\begin{array}{c} if\;HT(EV,V_{j})\geq T_{thr}\\ if\;HT(EV,V_{j})<T_{thr} \end{array}$$

- When the trust value $HT(EV,V_{j})$ exceeds or equals a predefined threshold $\left(T_{thr}\right)$, the sender vehicle is regarded as a “Trusted Vehicle”. The receiver/evaluator node will act upon the received message and broadcast the received trusted messages to other vehicles.
- The sender vehicle will be regarded as a “Malicious Vehicle“ and the received message will be discarded if the trust value $HT(EV,V_{j})$ is below the threshold ($T_{thr}$).
- The new trust value of the sender vehicle is stored in the trust database by the evaluator node.
- Malicious vehicles that continue to send false messages will be placed on the revocation list and sent to the TA.
- The TA revokes the pseudonyms of these vehicles and adds the revoked pseudonyms to the RPID-BC.

## 4. Security Analysis

(1) Source authentication: The VPID-BC and RPID-BC blockchains are adopted by the receiver/evaluator node (vehicle/RSU) to authenticate the sender vehicle by performing the PoP and PoA of the sender’s pseudo-identity ${PID}_{i}$.
(2) Message authentication: Our authentication scheme requires that each message a vehicle generates be signed before being sent to another vehicle or RSU. The receiver/evaluator node checks the signature in the received message to make sure that the received message has not been altered by attackers or malicious vehicles during transmission.
(3) Anonymity: The pseudonym ${PID}_{i}$ created by the TA is used by the vehicle to communicate with other vehicles/RSUs. When communicating, the pseudonym ${PID}_{i}$ keeps the real identity of the vehicle completely anonymous.
(4) Unlinkability: In our scheme, the sender vehicle $V_{i}$ transmits ${\{PID}_{i},{PK}_{i},M_{i},\sigma_{i},t_{i}\}$ to the neighboring vehicle/RSU. Messages from the same vehicle cannot be linked by attackers since the signature contains a random value. Unlinkability is therefore satisfied by the proposed scheme.
(5) Traceability and Revocability: When certain malicious vehicles are reported to the TA by RSUs, the TA can identify their real identity. Only the TA can determine the vehicle’s real identity from its pseudonym. The TA retrieves the vehicle’s real identity ${RID}_{i}={SID}_{i}⨁r_{i}{TA}_{pub}$ by obtaining $\left\{{PID}_{i},{SID}_{i},r_{i}\right\}$ from its database. The TA revokes the pseudonym ${PID}_{i}$ of the malicious vehicle and adds the revoked pseudonym ${PID}_{i}$ to the RPID-BC blockchain.
(6) Nonrepudiation: Due to the TA’s ability to link the pseudonym of a message to its real identity, no vehicle can deny signing a message.
(7) Anti-false message and combined attacks: The proposed framework takes advantage of V2V and V2I communication to detect false/bogus messages or thwart combined attacks. The proposed framework combines information from multiple sources, including beacon and event messages received from neighboring vehicles. This is utilized in order to compute the trustworthiness of the sender vehicle and to accurately detect false/bogus messages received from the sender vehicles due to malicious behavior. Thus, the proposed trust framework enables the receiver nodes to identify malicious vehicles and their false/bogus messages and eliminate them from the network.
(8) Resistance against attacks: Due to the signature, time-stamps, and random values of $l_{i}$, our scheme is resistant to message-tampering attacks, replay attacks, and man-in-the-middle attacks.

## 5. Performance Evaluation 

### 5.1. Efficiency Analysis

This section analyses our scheme’s efficiency (i.e., computation cost and communication overhead) and compares it to state-of-the-art schemes.

For schemes based on bilinear pairings, such as [37,38,39,40,41,42,43], we adopt the bilinear pairing $e:G_{1}\times G_{1}\to G_{2}$, where $G_{1}$ and $G_{2}$ are the additive cyclic group and multiplicative group, respectively, using the elliptic curve $\overset{-}{E}:y^{2}=x^{3}+xmod\overset{-}{p}$, where $\overset{-}{p}$ is a 512-bit prime number. A point $\overset{-}{P}$ on curve $\overset{-}{E}$ generates the additive group $G_{1}$ with order $\overset{-}{q}$ (a prime number of 160 bits). For the analysis of ECC-based schemes, we adopt an elliptic curve $E:y^{2}=x^{3}+ax+bmodp$ where $a,b\in Z_{q}^{*}$ and p,q are 160-bit primes. A point P on the elliptic curve E generates a cyclic additive group G with order q.

In Table 5, we describe different cryptographic operations along with their execution times [44]. 

#### 5.1.1. Computation Cost

When evaluating the computation cost, we consider the costs of signing a message, verifying individual signatures, and verifying n signatures. In our scheme, signing a message takes $T_{ecc\to sm}+T_{h}=0.4421$ ms, while verifying individual signatures takes $3T_{ecc\to sm}+2T_{ecc\to pa}+2T_{h}=1.3298$ ms. Thus, the total computation cost incurred to sign and verify an individual signature in our scheme is 1.7719 ms. In verifying n signatures, a receiver in our scheme needs $\left(n+2\right)T_{ecc\to sm}+\left(2n+2\right)T_{ecc\to pa}+2nT_{h}=0.4458n+0.8876$ ms. 

Comparing the proposed scheme to Kumar et al. [37], the proposed scheme’s percentage improvement in terms of signing a message, verification of an individual signature, and verification of n signatures (where n = 100) is calculated as $\frac{11.2564-0.4421}{11.2564}\times 100=96.07\%$, $\frac{30.7832-1.3298}{30.7832}\times 100=95.68\%$, and $\frac{9.5545n+21.2287-(0.4458n+0.8876)}{9.5545n+21.2287}\times 100=95.34\%,respectively$. The computation cost of other schemes and percentage improvement is calculated similarly, and presented in Table 6 and Table 7, respectively. 

**Figure 4:**
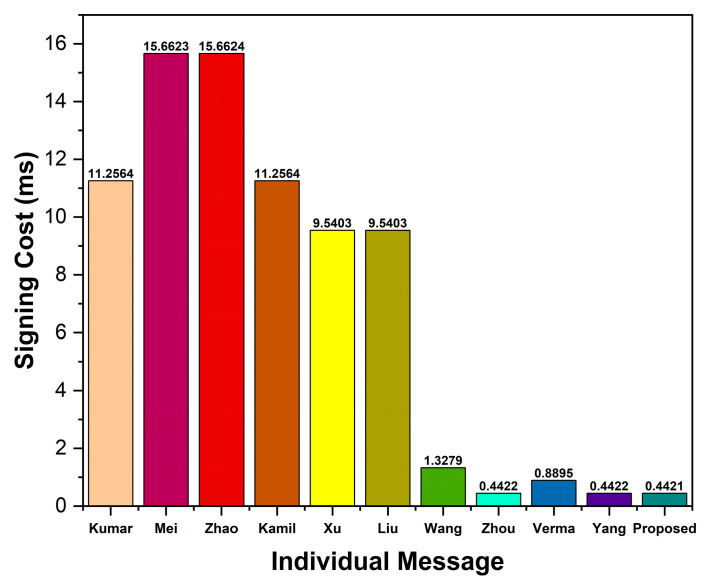
Signing cost of an individual message.

**Figure 5:**
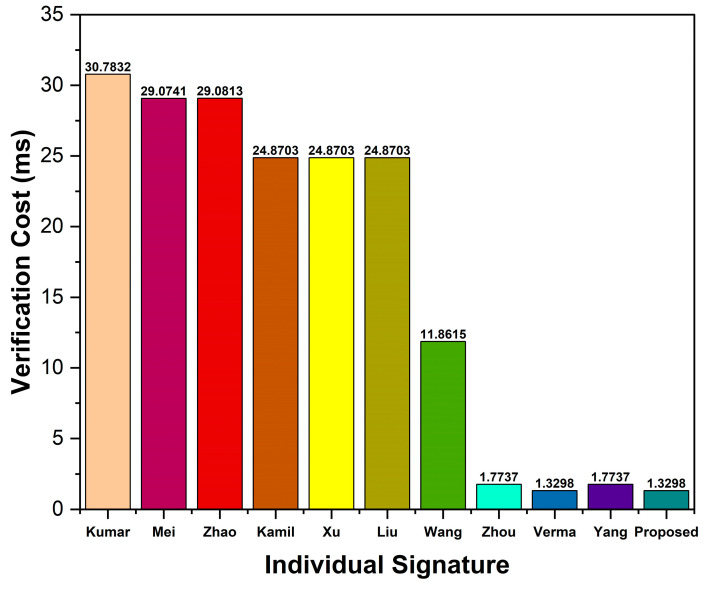
Verification cost of individual signature.

**Figure 6:**
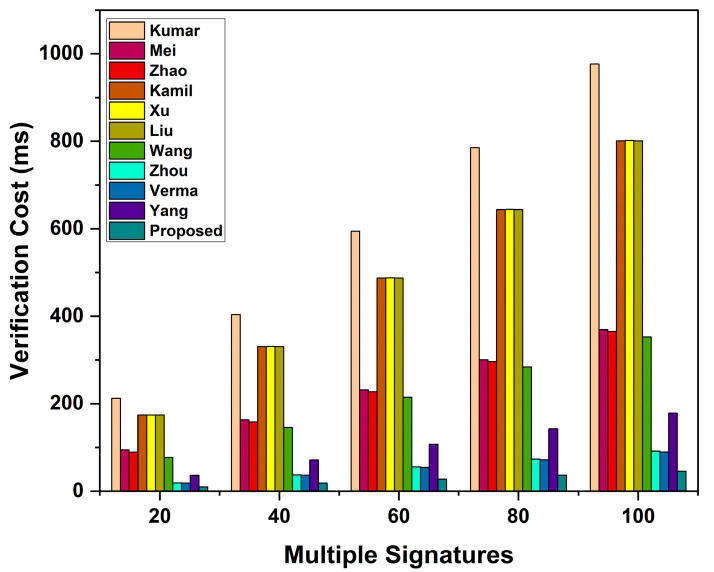
Verification cost of n signatures.

As shown in Table 6, the proposed scheme shows higher efficiency than bilinear-pairing-based schemes [37,38,39,40,41,42,43]. As compared to its related ECC-based scheme [45], the proposed scheme does not show much improvement in the signing of individual signatures. However, the proposed scheme depicts a 25% reduction in verifying costs over the scheme [45]. Additionally, our scheme shows higher efficiency than the related ECC-based schemes [46,47]. Figure 4, Figure 5 and Figure 6 show the signing cost of an individual message, the verification cost of an individual signature, and the verification cost of n signatures, respectively. 

Based on the comparative analysis (see Table 6 and Table 7, and Figure 4, Figure 5 and Figure 6), we show that our proposed scheme has the lowest computation cost than other ECC- and bilinear-pairing-based schemes. Consequently, the proposed scheme has been proven to be more efficient than other schemes. Thus, the proposed scheme is more feasible for practical VANET applications.

#### 5.1.2. Communication Overhead

The communication overhead of our scheme is analyzed and compared with that of other schemes [37,38,39,40,41,42,43,44,45,46,47]. According to Ref. [48], the sizes of the elements used for the analysis of communication overhead are listed in Table 8. 

We evaluate communication overhead by summing the transmission overhead caused by pseudo-identity ${PID}_{i}$, public key ${PK}_{i}$, timestamp $t_{i}$, and signature $\sigma_{i}$. We do not include the message $M_{i}$ based on the assumption that it is a constant factor. In our scheme, a sender broadcasts a message ${\{PID}_{i},{PK}_{i},M_{i},\sigma_{i},t_{i}\}$ where pseudo-identity is ${PID}_{i}=({VID}_{i},T_{i})$, public key is ${PK}_{i}$, signature is $\sigma_{i}=(L_{i},D_{i})$, and $t_{i}$ is the timestamp. ${PK}_{i},L_{i}\in G$,${VID}_{i},$ $D_{i}\in Z_{q}^{*}$, and $T_{i},t_{i}$ are timestamps. Thus, our scheme incurs 2$\left|G\right|+2\left|Z\right|+2\times 4=128$ bytes as the total communication overhead. Similarly, the communication overhead of other schemes has been calculated and is presented in Table 9. Table 9 shows that our scheme has the lowest communication overhead compared to other schemes. Therefore, the proposed scheme is more efficient than the baseline schemes, and can thus support the transmission of messages between vehicles and RSUs more securely and effectively.

### 5.2. Simulation-Based Analysis

#### 5.2.1. Simulation Setup

Our proposed trust framework was evaluated using Veins, a popular open-source framework for simulating vehicular networks [49,50]. Veins integrates SUMO (the road traffic simulator) [51,52] and OMNeT++ (the network simulator) [53]. In an OMNeT++ simulation, node movement corresponds to vehicle movement in the SUMO road traffic simulator. 

**Figure 7:**
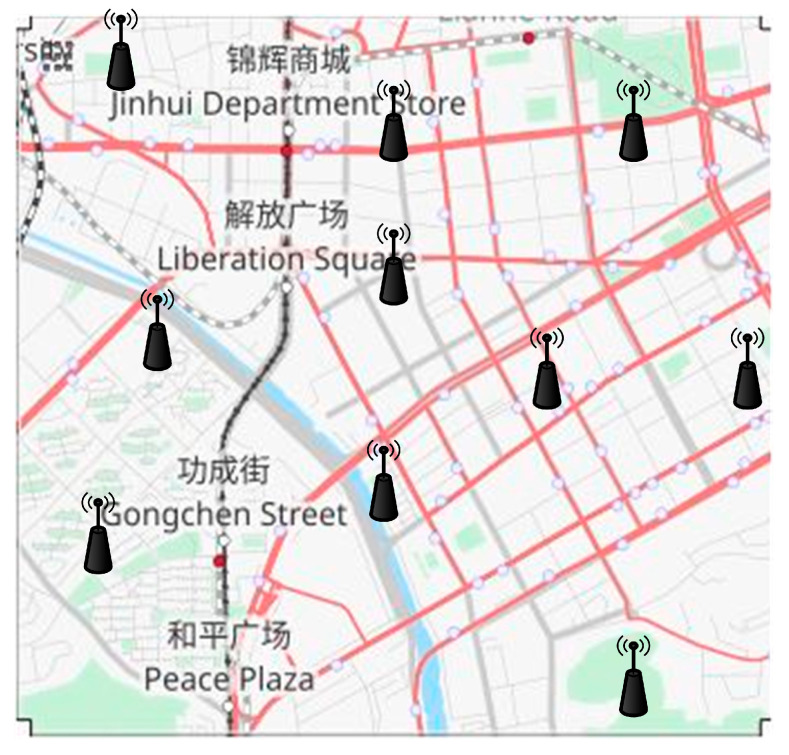
Network map.

Figure 7 illustrates a real map extracted from OpenStreetMap [54,55]. Our proposed trust framework operates on legitimate vehicles and RSUs in our simulations, allowing them to verify the authenticity and trustworthiness of the network events. Vehicles may encounter each other over and over. The duration of an event is 20 s.

The initial node placement varies for each run in each simulation scenario since each run uses a different random seed. Each experimental result for each simulation scenario is the average over the 20 runs. The parameters used in the simulation scenarios are listed in Table 10.

#### 5.2.2. Performance Evaluation Metrics

Our proposed trust framework is evaluated using three metrics: Precision, Recall, and F-Measure. The Precision, Recall, and F-Measure are defined as follows:

- Precision: Precision is the proportion of the relevant nodes that were successfully identified as malicious vehicles over the total number of nodes that were both correctly and incorrectly identified as malicious vehicles.

(14) $$Precision=\frac{TP}{TP+FP}$$

TP is the number of malicious vehicles correctly identified, and FP is the number of vehicles incorrectly classified as malicious.

- Recall: Recall is the proportion of the number of vehicles correctly classified as malicious over the total number of malicious vehicles.

(15) $$Recall=\frac{TP}{TP+FN}$$

FN is the number of malicious vehicles that were incorrectly classified as legitimate vehicles.

- F-Measure: F-Measure indicates how accurate the trust scheme is at identifying malicious vehicles and their false messages based on the weighted average of precision and recall. As a result, F-Measure provides a measure for the trust model’s accuracy. The higher the F-Measure, the higher the accuracy of the trust model.

(16) $$F-Measure=2\times \frac{Precision\times Recall}{Precision+Recall}$$

- End-To-End Delay: We measure the latency in terms of end-to-end delay, i.e., the time it takes a message to arrive at the receiver from its sender. End-to-end delay is calculated as below:
  (17) $$End-to-End\;Delay=\sum (T_{Arrival}-T_{Sent})$$
  where $T_{arrival}$ is the time a message arrived at its receiver and $T_{sent}$ refers to the time the message was sent from the sender.

#### 5.2.3. Performance Evaluation under Different Contexts

In this section, we describe the contexts in which trust model efficiency is to be evaluated. We evaluate and compare the performance of the proposed trust framework to those of Gao et al. [25] and DUEL [26] in three contexts/scenarios: vehicle density (different number of legitimate vehicles), different vehicle speeds, and different percentages of malicious vehicles, respectively.

*Context 1: Vehicle Density*

In general, traffic conditions affect trust scheme accuracy. We evaluate and compare the adaptability of these schemes to various vehicle densities. Low traffic density makes it difficult for the evaluator node to accurately evaluate the target vehicle’s trust due to the lack of sufficient information. Low-density, sparsely connected networks cause communication links to frequently disconnect. Therefore, there are no frequent interactions between vehicles. Vehicles may not receive enough data in low-density environments to make reliable trust evaluations, which leads to unreliable trust decisions. Under scenarios with low traffic density, the trust scheme only has a limited number of data sources to evaluate and update the sender’s vehicle trust. Under high-density scenarios, communication becomes more effective and data can be disseminated more reliably. The dense vehicle distribution will maintain a high number of interactions between vehicles. More interactions will allow frequent message exchange for data dissemination. Through these interactions, trust models can gain direct interactions, indirect recommendations, and reliable multi-hop data transmission. However, due to data redundancy, communication overhead may occur from extremely high densities. 

*Context 2: Vehicle Speed*

Vehicle Speed (velocity) represents a vehicle’s mobility, and the faster a vehicle moves, the harder it is to gather enough data from other vehicles because highly mobile vehicles have a limited amount of communication time. Due to high vehicle mobility, networks are sparsely connected, disconnections are frequent, and communication links are error-prone. High mobility typically results in frequent disconnections, which lowers data delivery ratios. As mobility decreases, the efficiency of communication increases, data can be disseminated more reliably, and vehicles can have more interactions to collect sufficient information for trust evaluation.

*Context 3: Percentage of Malicious Vehicles*

To evaluate how efficiently the trust model counteracts attacker behavior, we added malicious vehicles into the network. At first, we introduced 10% of the network’s malicious vehicles and these malicious vehicles polluted the network with false messages or launched combined attacks. We gradually increased the proportion of malicious vehicles until it reached 50% to evaluate the effectiveness of the trust model in detecting false/bogus messages and malicious vehicles in the network. 

#### 5.2.4. Simulation Results and Discussion

The efficiency of our trust management framework was compared with two baseline trust management schemes, i.e., Gao et al. [25], and DUEL [26].

##### Influence of Vehicle Density on Trust Schemes 

**Figure 8:**
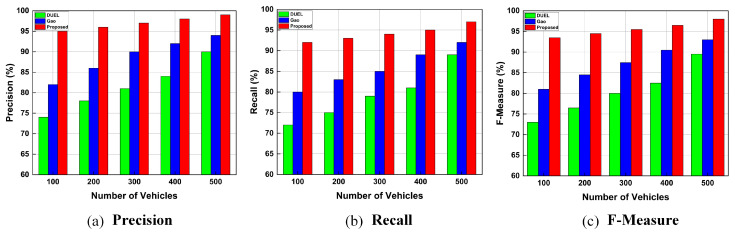
Influence of vehicle density on trust schemes.

The proposed trust management framework was evaluated by varying the network’s number of legitimate vehicles. The density of the vehicles was increased from 100 to 500 vehicles. Additionally, 10% of vehicles were chosen as malicious vehicles that consistently send bogus/false messages. Figure 8 depicts the precision, recall, and F-Measure of the proposed trust management framework, Gao, and DUEL schemes, when the network has different densities of legitimate vehicles. The proposed trust framework performed better than the two baseline schemes and guaranteed the dissemination of trusted messages throughout the network, and considerably enhanced precision, recall, and F-Measure even when there were fewer legitimate vehicles. The higher precision, recall, and F-Measure indicate the higher efficiency of the proposed framework in detecting malicious vehicles and their bogus messages. The reason for the better performance of our framework is that it adopts multiple sources of information (information from other vehicles, RSUs, and beacon messages) to efficiently evaluate the trustworthiness of the sender vehicles and the messages they generate in low-density traffic scenarios. As the number of legitimate vehicles in the network increases, more trustworthy/legitimate vehicles will become available, which will enable more trusted messages to be distributed throughout the network, thereby increasing the probability for a receiver vehicle to receive trustworthy data from others. Therefore, when more legitimate vehicles are available in the network, the precision, recall, and F-measure increase. 

The proposed trust framework outperforms existing baseline trust schemes and efficiently detects malicious vehicles and their false messages when the number of legitimate vehicles is low or high. The proposed framework achieved more than 90% accuracy in terms of F-Measure, while the baseline schemes achieved 81% and 72%, respectively, demonstrating the high efficiency of our framework. As indicated, the higher precision, recall, and F-Measure indicates a higher ability of the trust scheme to identify malicious vehicles and their bogus messages and revoke them from the vehicular network under various densities of legitimate vehicles. The better performance of our trust framework is attributed to the authentication module, which ensures that messages originate from registered vehicles and are not modified during transmission. Moreover, our framework adopts multiple sources of information (information from other vehicles, RSUs, and beacon messages) to efficiently identify those malicious vehicles and their bogus messages. Therefore, as shown in Figure 8, the proposed trust framework outperformed the Gao and DUEL schemes based on precision, recall, and F-Measure values even when the legitimate vehicle density varied.

##### Influence of Vehicle Speed on Trust Schemes

**Figure 9:**
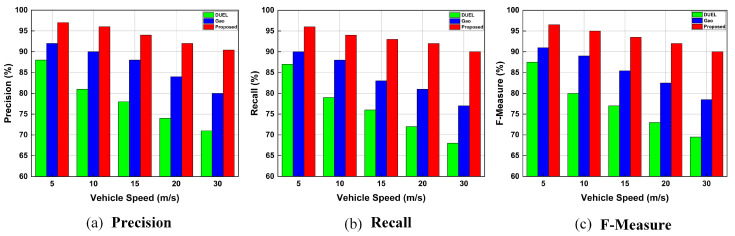
Influence of vehicle speed on trust schemes.

Figure 9 demonstrates the comparison of the precision, recall, and F-Measure of the proposed trust framework, Gao, and DUEL schemes when the vehicles are moving at different speeds. Figure 9a–c illustrate that the precision, recall, and F-Measure of the proposed trust framework, and other baseline schemes are affected by vehicle speed. This effect increased when the vehicles were moving faster. This is because the average communication time between vehicles reduces as vehicle speed increases. Short communication time between vehicles results in fewer interactions (and therefore fewer data exchanges), which reduces the trust scheme’s accuracy and efficiency for detecting malicious vehicles and their bogus messages. However, the proposed trust framework still significantly improves the precision, recall, and F-Measure when vehicles are moving at high speeds due to the numerous parameters/sources of information used for trust evaluation. By utilizing the maximum amount of available information to increase the accuracy of trust evaluation, the proposed trust framework mitigates the effects of insufficient information from neighboring vehicles during trust evaluation. 

Figure 9a–c show that the proposed trust framework achieves a higher precision, recall, and F-Measure compared to the other baseline schemes. The accuracy of the proposed trust framework, Gao, and DUEL schemes, in terms of F-Measure, is 90%, 78%, and 69% at the highest speed of vehicles (30 m/s), respectively. The higher accuracy of the proposed trust framework indicates its better ability to detect and revoke malicious vehicles and their bogus messages when vehicles are moving at high speeds. 

When vehicles move at low speeds, the evaluator node will experience sufficient direct interactions with the sender vehicle and nearby vehicles due to the longer communication time between them. As a result, the recall, precision, and F-Measure are all increased. The higher precision, recall, and F-Measure indicate a higher ability and accuracy of the proposed trust framework to identify malicious vehicles and their false/bogus messages when vehicles move at low speeds. The results further show that when the vehicles are moving at various speeds, the proposed trust framework outperforms both the Gao and DUEL schemes under various vehicle speeds. 

##### Influence of Different Percentages of Malicious Vehicles 

**Figure 10:**
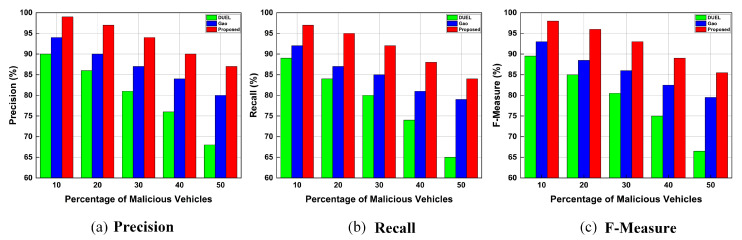
Influence of malicious vehicles percentage on trust schemes.

Figure 10 illustrates the precision, recall, and F-Measure for the proposed trust framework, Gao, and DUEL schemes when the network has different percentages of malicious vehicles. The malicious vehicles carry out combined attacks in which the attacker creates bogus messages individually, performs opinion alteration, on–off patterns, or colludes with other malicious vehicles to share bogus messages within the network. The percentage of attackers was increased from 10% to 50%. Figure 10 illustrates that high precision, recall, and F-Measure values can be achieved when fewer attackers perform combined attacks on the network. However, precision, recall, and F-Measure decreased when the number of attackers increased in the network, because attackers performing combined attacks pollute the network with bogus messages, and malicious vehicles prevent legitimate vehicles from exchanging correct information. Since the information provided by malicious vehicles is false, malicious vehicles can compromise trust evaluation by colluding. Thus, legitimate vehicles cannot distinguish between legitimate and bogus messages. In comparison with baseline schemes, our proposed trust framework ensures high precision, recall, and F-Measure, showing that our proposed framework can cope and deal effectively with combined attacks even when the number of attackers is high. The reason for achieving this accuracy is that the proposed trust framework incorporates the authentication module for early detection of malicious vehicles and their bogus messages, and adopts the trust evaluation modules that utilize information from multiple sources (information from other vehicles, RSUs, and beacon messages) for accurate trust evaluation and better identification and revocation of malicious vehicles and the bogus messages they generate. Thus, our trust framework can quickly detect malicious vehicles and their false/bogus messages. The bogus messages are revoked and cannot be disseminated further. As a result, legitimate vehicles can receive trusted messages even when malicious vehicles are present in the network. For a network with 50% malicious vehicles, our trust framework guarantees an F-Measure (accuracy) of 85%, whereas the baseline trust schemes, Gao and DUEL, achieve an accuracy of less than 79%, and 66%, respectively. The high accuracy of our framework indicates its superiority in identifying and revoking malicious vehicles and thwarting their attacks. Additionally, Figure 10a–c show that the proposed trust framework outperforms baseline trust schemes in terms of achieving higher precision, recall, and F-Measure (accuracy) under various proportions of malicious vehicles in the network. 

##### End-to-End Delay

**Figure 11:**
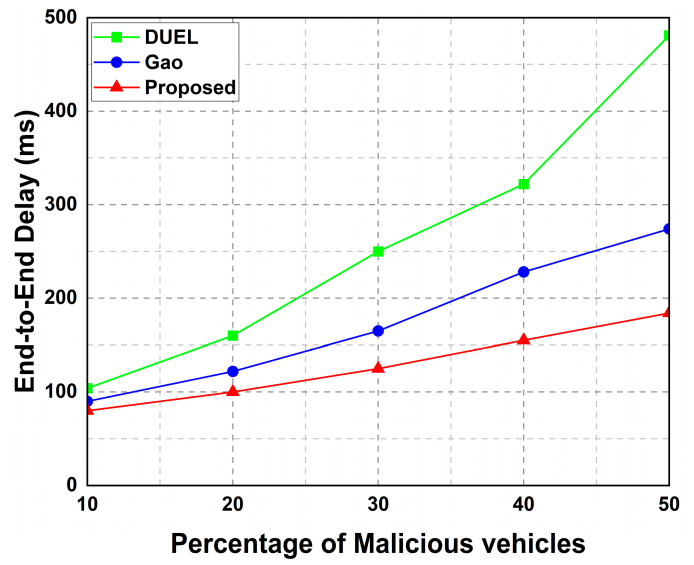
End-to-end delay.

We evaluated the latency in terms of end-to-end (E2E) delay. A low end-to-end delay is essential because VANET applications are delay-sensitive. As shown in Figure 11, the addition of malicious vehicles increased the end-to-end delay in all schemes. However, even when the network contained a high number of malicious vehicles, the proposed framework had the lowest latency compared to the other baseline schemes. 

The proposed framework outperforms other schemes because of its lightweight trust computation, lightweight messages, and low communication overhead. This means that the messages were processed and transmitted in a short time. Thus, our framework can immediately identify malicious vehicles and their false/bogus messages, enabling other vehicles to receive trusted information on time.

## 6. Conclusions

In this paper, we propose a blockchain-assisted privacy-preserving and context-aware trust management framework to secure V2V and V2I communications in VANETs by integrating a blockchain-assisted privacy-preserving authentication scheme and a context-aware trust management scheme. The proposed framework enables the receiver node (vehicle/RSU) to authenticate and evaluate the trustworthiness of the sender vehicles and their messages and identify and revoke the bogus messages and the malicious vehicles generating them. Additionally, we incorporated the blockchain into the proposed framework to facilitate efficient and distributed vehicle authentication and to enhance the security and privacy of the network. The proposed framework consists of several modules and exploits information from several sources (beacon messages, event messages, other vehicles, and RSUs) to enable the receiver node to evaluate the trustworthiness of the sender vehicles and their messages, and efficiently identify and revoke the malicious vehicles and their bogus messages in various contexts of VANETs. The proposed framework satisfies the trust, efficiency, security, privacy, and dynamic requirements of VANETs.

Based on the security analysis, efficiency analysis, and experimental results, the proposed framework is more efficient in terms of having a lower computational cost and communication overhead, lower end-to-end delay, and higher precision, recall, and F-measure (accuracy) than baseline schemes. Therefore, the proposed framework is secure, efficient, and feasible for VANETs.

In future work, we will integrate machine learning techniques into the proposed framework to adjust the trust threshold to be dynamic and to derive more representative context features for accurate representation, and for precisely detecting malicious vehicles and their false messages. This will further enhance the overall performance of our framework.

## Figures and Tables

**Table 1:** Comparison of our trust framework with other trust schemes.

Scheme	Authentication	Privacy Preserving	Other Security Requirements (Non-Repudiation and Unlinkability)	Context Awareness (Adapting to Various Contexts)				Role-Based Nodes
				Adapting to Variations in Malicious Vehicles Density		Adapting to Variations in Legitimate Vehicle Density	Adapting to Variations in Vehicles Speed	
Liu et al. [22]	Yes	Yes	No	No				No
Liu et al. [23]	Yes	Yes	Yes	High		No	Normal	Yes
Guo et al. [24]	No	No	No	High		No		No
Gao et al. [25]	No	No	No	Normal		Normal	High	No
Bhargava and Verma [26]	No	No	No	Normal		Normal	Normal	No
Liu et al. [27]	Yes	No	Non-repudiation is achieved but unlinkability is not provided	Normal		No		Yes
Inedjaren et al. [28]	No	No	No	High		No		No
Ghaleb et al. [29]	No	No	No	Normal		Normal	High	No
Ahmad et al. [30]	No	No	No	High		No	Normal	Yes
Ghaleb et al. [31]	No	No	No	No		High	Normal	No
Rehman et al. [32]	No	No	No	High		Normal	No	Yes
Ghajar et al. [33]	No	No	No	No				No
Chukwuocha et al. [34]	No	No	No	No				No
Hasrouny et al. [35]	Yes	Yes	Non-repudiation is achieved but unlinkability is not provided	No				No
**Scheme**	**Adopts Blockchain** **(Secure and Efficient Data Storage)**	**Communication Scenario**		**Traceability and Revocation of Malicious Vehicles**	**Efficiency** **(Computational Cost and Communication Overhead)**		**Resistance to Attacks**	
		**V2V**	**V2I**					
Liu et al. [22]	No	Yes	No	No	Efficient		False-message attacks, replay attacks, message-tampering attack	
Liu et al. [23]	No	Yes	No	Malicious vehicles can be traced but no revocation	Efficient in terms of communication overhead but the computation cost is not provided		Malicious vehicles	
Guo et al. [24]	No	Yes	No	No	No		False-message attacks	
Gao et al. [25]	No	Yes	No	No	No		False-message attacks, message-tampering attacks, message-dropping attacks, and opinion-tampering attacks	
Bhargava and Verma [26]	No	Yes	No	No	No		False-message attacks, message- tampering attacks, and message-dropping attacks	
Liu et al. [27]	No	Yes	No	Yes	No		False-message attacks, opinion-tampering attacks	
Inedjaren et al. [28]	Yes	Yes	No	No	No		Message-dropping attacks	
Ghaleb et al. [29]	No	Yes	No	No	No		False-message attacks	
Ahmad et al. [30]	No	Yes	No	No	No		Message-tampering attacks, message-delaying attacks	
Ghaleb et al. [31]	No	Yes	No	No	No		Malicious vehicles	
Rehman et al. [32]	No	Yes	No	No	No		Malicious vehicles	
Ghajar et al. [33]	Yes	Yes	No	No	No		False-message attacks	
Chukwuocha et al. [34]	Yes	Yes	No	No	No		False-message attacks	
Hasrouny et al. [35]	No	Yes	No	Yes	No		Malicious vehicles	

**Table 2:** Notations.

Notation	Description
${RSU}_{i}$	Roadside unit i
$V_{i}$	Vehicle $i^{th}$
G	ECC-based cyclic additive group
$G_{1}$	Bilinear pairing-based cyclic additive group
P	Generator of G
E	An elliptic curve
p,q	Two large prime numbers
${RID}_{i}$	Vehicle $V_{i}$’s real identity
${PID}_{i}$	Vehicle $V_{i}$’s pseudonym
${TA}_{pub},$ s	Public and secret master key pair of the TA
R,y	Public and private parameters generated by TA
${PK}_{i},{SK}_{i}$	Public and private keys of $V_{i}$
${PU}_{i},{PR}_{i}$	${RSU}_{i}$’s public and private keys
${ppk}_{i}$	Vehicle $V_{i}$ partial private key
h1,h2,h3,h4	Cryptographic one-way hash functions
$T_{i},t_{i}$	Validity period of ${PID}_{i}$ and the message timestamp
$M_{i}$	A message from $V_{i}$
$\sigma_{i}$	Signature from $V_{i}$ on message $M_{i}$
⨁	Exclusive OR operator

**Table 3:** Beacon messages sent by the neighboring vehicles of vehicle V_B_.

V_B_					
	Pseudo-Identity (PID)	$M_{1}(beacon)$			
		$\mathrm{Location}\;\left(x_{1},y_{1}\right)$		$\mathrm{Speed}\;(v_{1})$	$\mathrm{Direction}\;(d_{1})$
		$\mathrm{Lat}\;(x_{1})$	$\mathrm{Long}\;(y_{1})$		
V_A_	PID_A_	22.421400	121.490800	20	W
V_C_	PID_C_	22.421600	121.490600	21	W
V_D_	PID_D_	22.421200	121.490100	24	E

**Table 4:** Event message sent by vehicle V_A_ to the neighboring vehicles and RSU.

V_A_						
Pseudo-Identity (PID)	$M_{1}(event)$					
	Vehicle Role $\left(V_{Role}\right)$	$\mathrm{Type}\;\mathrm{of}\;\mathrm{Event}\;(E_{Type})$	$\mathrm{Location}\;\mathrm{of}\;\mathrm{Event}\;(E_{x},E_{y})$		Location of V_A_ $\left(L_{x},L_{y}\right)$	
PID_A_	OV	Traffic jam	22.421200	121.490700	22.21100	121.490600

**Table 5:** Notation of cryptographic operations and their execution time.

Notation	Operation	Execution Time (ms)
$T_{bp}$	Bilinear pairing	4.2110
$T_{bp\to sm}$	Bilinear-pairing-based scalar multiplication	1.7090
$T_{bp\to pa}$	Bilinear-pairing-based point addition	0.0071
$T_{mtp}$	A map-to-point hash function	4.406
$T_{ecc\to sm}$	ECC-based scalar multiplication	0.4420
$T_{ecc\to pa}$	ECC-based point addition	0.0018
$T_{h}$	A secure hash function	0.0001

**Table 6:** Comparative analysis of computation costs.

Scheme	Signing Cost (ms)	Verification Cost of Individual Signature (ms)	Verification Cost of n Signatures (ms)
Kumar et al. [37]	$4T_{bp\to sm}+2T_{bp\to pa}+T_{mtp}+2T_{h}=11.2564$	$4T_{bp}+3T_{bp\to sm}+2T_{mtp}+2T_{h}=30.7832$	$4T_{bp}+3nT_{bp\to sm}+3\left(n-1\right)T_{bp\to pa}+\left(n+1\right)T_{mtp}+2nT_{h}=9.5545n+21.2287$
Mei et al. [38]	$4T_{bp\to sm}+2T_{bp\to pa}+2T_{mtp}+T_{h}=15.6623$	$4T_{bp}+2T_{bp\to sm}+2T_{mtp}+T_{h}=29.0741$	$4T_{bp}+2nT_{bp\to sm}+\left(2n-2\right)T_{bp\to pa}+2T_{mtp}+nT_{h}=3.4323n+25.6418$
Zhao et al. [39]	$4T_{bp\to sm}+2T_{bp\to pa}+2T_{mtp}+2T_{h}=15.6624$	$4T_{bp}+2T_{bp\to sm}+T_{bp\to pa}+2T_{mtp}+2T_{h}=29.0813$	$4T_{bp}+2nT_{bp\to sm}+\left(4n-3\right)T_{bp\to pq}+2T_{mtp}+2{nT}_{h}=3.4466n+20.2906$
Kamil et al. [40]	$4T_{bp\to sm}+2T_{bp\to pa}+T_{mtp}+2T_{h}=11.2564$	$3T_{bp}+2T_{bp\to sm}+T_{bp\to pa}+2T_{mtp}+2T_{h}=24.8703$	$3T_{bp}+2nT_{bp\to sm}+\left(2n-1\right)T_{bp\to pa}+\left(n+1\right)T_{mtp}+nT_{h}=7.8383n+17.0319$
Xu et al. [41]	$3T_{bp\to sm}+T_{bp\to pa}+T_{mtp}+2T_{h}=9.5403$	$3T_{bp}+2T_{bp\to sm}+2T_{mtp}+T_{bp\to pa}+2T_{h}=24.8703$	$3T_{bp}+\left(3n-2\right)T_{bp\to pa}+2nT_{bp\to sm}+\left(n+1\right)T_{mtp}+2nT_{h}=7.8455n+17.0248$
Liu et al. [42]	$3T_{bp\to sm}+T_{bp\to pa}+T_{mtp}+2T_{h}=9.5403$	$3T_{bp}+2T_{bp\to sm}+2T_{mtp}+T_{bp\to pa}+2h=24.8703$	$3T_{bp}+2nT_{bp\to sm}+\left(2n-1\right)T_{bp\to pa}+\left(n+1\right)T_{mtp}+2nT_{h}=7.8384n+17.0319$
Wang et al. [43]	$3T_{bp\to sm}+T_{bp\to pa}+T_{h}=1.3279$	$2T_{bp}+2T_{bp\to sm}+3T_{bp\to pa}+2T_{h}=11.8615$	$2T_{bp}++2nT_{bp\to sm}+3nT_{bp\to pa}+2nT_{h}=3.4395n+8.422$
Zhou et al. [45]	$T_{ecc\to sm}+2T_{h}=0.4422$	$4T_{ecc\to sm}+3T_{ecc\to pa}+3T_{h}=1.7737$	$\left(2n+2\right)T_{ecc\to m}+\left(3n+3\right)T_{ecc\to pa}+3nT_{h}=0.9056n+0.8911$
Verma et al. [46]	${2T}_{ecc\to sm}+3T_{ecc\to pa}+T_{h}=0.8895$	$3T_{ecc\to sm}+2T_{ecc\to pa}+2T_{h}=1.3298$	$\left(2n+2\right)T_{ecc\to sm}+(n+2)T_{ecc\to pa}+2nT_{h}=0.886n+0.8876$
Yang et al. [47]	$T_{ecc\to sm}+2T_{h}=0.4422$	$4T_{ecc\to sm}+3T_{ecc\to pa}+3T_{h}=1.7737$	$\left(4n+1\right)T_{ecc\to sm}+\left(5n+3\right)T_{ecc\to pa}+(6n{+1)T}_{h}=1.7776n+0.4475$
The proposed scheme	$T_{ecc\to sm}+T_{h}=0.4421$	$3T_{ecc\to sm}+2T_{ecc\to pa}+2T_{h}=1.3298$	$\left(n+2\right)T_{ecc\to sm}+\left(2n+2\right)T_{ecc\to pa}+2nT_{h}=0.4458n+0.8876$

**Table 7:** The proposed scheme’s percentage improvement over other schemes.

Schemes	Signing a Message (%)	Verifying Individual Signature (%)	Verifying n Signatures (n=100)
Kumar et al. [37]	96.07	95.68	95.34
Mei et al. [38]	97.18	95.43	87.67
Zhao et al. [39]	97.18	95.43	87.54
Kamil et al. [40]	96.07	94.65	94.32
Xu et al. [41]	95.37	94.65	94.33
Liu et al. [42]	95.37	94.65	94.32
Wang et al. [43]	66.71	88.79	87.1
Zhou et al. [45]	0.02	25.03	50.28
Verma et al. [46]	50.30	0.00	49.19
Yang et al. [47]	0.02	25	74.49

**Table 8:** Sizes of various elements.

Type of Element	Description	Size (Bytes)
$\left|G_{1}\right|$	An element of a group $G_{1}$	128
$\left|G\right|$	An element of a group G	40
$\left|Z_{q}^{*}\right|$	An element of a finite field $Z_{q}^{*}$	20
$\left|t_{i}\right|$	Time-stamp	4

**Table 9:** Comparison of communication overhead.

Scheme	Single Message (Bytes)	n Messages (Bytes)
Kumar et al. [37]	536	536n
Mei et al. [38]	540	540n
Zhao et al. [39]	388	388n
Kamil et al. [40]	540	540n
Xu et al. [41]	404	404n
Liu et al. [42]	404	404n
Wang et al. [43]	792	792n
Zhou et al. [45]	208	208n
Verma et al. [46]	220	220n
Yang et al. [47]	164	164n
The proposed scheme	128	128n

**Table 10:** Simulation parameters.

Parameter	Value	
Simulation Area	3 km × 3 km	
Simulation Time	1000 s	
Number of RSUs	10	
Number of Vehicles	100, 200, 300, 400, 500	
Role-Based Vehicles (%)	AV	10
	PS	20
	OV	70
Vehicle Speed	5 m/s, 10 m/s, 15 m/s, 20 m/s, 30 m/s	
Malicious Vehicles (%)	10, 20, 30, 40, 50	
Network Protocol	WAVE	
MAC Protocol	IEEE 802.11p	
Transmission Range	300 m	
Trust Threshold	0.5	
Initial Trust	0.3	

## Data Availability

Not applicable.
